# Supplementary material for: Discovery and application of insertion-deletion (INDEL) polymorphisms for QTL mapping of early life-history traits in Atlantic salmon
Source: BMC Genomics. 2010 Mar 8;11:156. doi: 10.1186/1471-2164-11-156 (PMC2838853; doi:10.1186/1471-2164-11-156)
Supplement: Additional file 2 — Information on developed 76 locus single-run INDEL panel in Atlantic salmon. Information on fluorescence labeling, primer concentrations, PCR pooling and links to alignments, INDEL motifs and GENESCAN (Burge and Karlin 1997) predictions of genes/exons are available in html format. [file 1471-2164-11-156-S2.ZIP › Additionalfile2/snpsummary9552.html]

```
Cluster 3198 Contig 1

prev  Summary    Contig List  next
```

Size of Consensus sequence = 2174

Number of sequences = 6

Minimum redundancy = 2

Key

A gi|117829932|gb|EG902628.1|EG902628 EST\_ssal\_evf\_2938 ssalevf mixed\_tissue Salmo salar cDNA Salmo salar cDNA clone ssal\_evf\_502\_084\_fwd 3', mRNA sequence  
B gi|117829943|gb|EG902639.1|EG902639 EST\_ssal\_evf\_2939 ssalevf mixed\_tissue Salmo salar cDNA Salmo salar cDNA clone ssal\_evf\_502\_084\_rev 5', mRNA sequence  
C gi|117424905|gb|EG757129.1|EG757129 EST\_ssal\_sjb\_5819 ssalsjb mixed\_tissue Salmo salar cDNA Salmo salar cDNA clone ssal\_sjb\_013\_196\_rev 5', mRNA sequence  
D gi|89856688|gb|DY712811.1|DY712811 EST\_ssal\_rgb2\_68550 ssalrgb2 mixed\_tissue Salmo salar cDNA Salmo salar cDNA clone ssal\_rgb2\_611\_117\_fwd 3', mRNA sequence  
E gi|117424906|gb|EG757130.1|EG757130 EST\_ssal\_sjb\_5820 ssalsjb mixed\_tissue Salmo salar cDNA Salmo salar cDNA clone ssal\_sjb\_013\_196\_fwd 3', mRNA sequence  
F gi|89856687|gb|DY712810.1|DY712810 EST\_ssal\_rgb2\_68549 ssalrgb2 mixed\_tissue Salmo salar cDNA Salmo salar cDNA clone ssal\_rgb2\_611\_117\_rev 5', mRNA sequence

3 SNPs detected

A B C D E F  cosegregation weighted

258 - - C C . .   3/3 66.67
259 - - T T . .   3/3 66.67
365 T T G G . .   3/3 66.67
